# Pain, motor function and health-related quality of life in children with cerebral palsy as reported by their physiotherapists

**DOI:** 10.1186/1471-2431-14-192

**Published:** 2014-07-27

**Authors:** Marta Badia, Inmaculada Riquelme, Begoña Orgaz, Raquel Acevedo, Egmar Longo, Pedro Montoya

**Affiliations:** 1 INICO, Faculty of Psychology, University of Salamanca, Salamanca, Spain; 2 Research Institute on Health Sciences (IUNICS), University of Balearic Islands, Carretera de Valldemossa km 7.5, 07122 Palma, Spain; 3 Department of Nursing and Physiotherapy, University of Balearic Islands, Palma, Spain; 4 ASPACE-Castile Leon, Faculty of Psychology, University of Salamanca, Salamanca, Spain

**Keywords:** Pain, Cerebral palsy, Quality of life, Motor function, Adolescents, Child development

## Abstract

**Background:**

Children and adolescents with cerebral palsy suffer from higher levels of pain than their peers without disability. The aim of this study was to explore the impact of pain on health-related quality of life and motor function in individuals with cerebral palsy as reported by health professionals.

**Methods:**

Cross-sectional study carried out in Associations for Care of Individuals with Cerebral Palsy and Related Disabilities (ASPACE) in Balearic Islands and Castile Leon (Spain). Thirty-five physiotherapists rated pain, health-related quality of life and motor function in 91 children and adolescents with cerebral palsy [8-19y]. A semi-structured interview was used to collect demographic and clinical data according with the Study of Participation of Children with Cerebral Palsy Living in Europe (SPARCLE).

**Results:**

Physiotherapists reported that 51% of individuals with cerebral palsy suffered from pain. Physiotherapists also perceived that pain in individuals with cerebral palsy was responsible for reductions of psychological but not physical domains of health-related quality of life. According with physiotherapists’ estimations, motor impairment scores were not correlated with pain scores in individuals with cerebral palsy, but they were significantly associated with physical and autonomy domains of health-related quality of life.

**Conclusions:**

These findings highlighted the importance of assessing and providing interventions for pain relief in persons with cerebral palsy even at an early age.

## Background

There is a growing recognition that pain is a significant problem in children and adolescents with cerebral palsy (CP). More than 50% of children with CP suffer pain from moderate to severe intensity on a daily basis and at multiple body locations [1-5]. Pain usually interferes with physical function, school, daily care activities, sleep and mental health [6-9]. Moreover, chronic pain may negatively affect health-related quality of life (HRQOL), limiting life satisfaction [4,10-12] and health perception in CP [9,13]. In this sense, HRQOL is considered a multidimensional construct that embraces several domains of physical and psychological well-being [13-17]. Moreover, suffering from persistent pain seems to reduce emotional, social and physical domains of HRQOL in children with CP [14].

Although self-report is the gold standard for pain and HRQOL assessment in pediatric settings, it may be difficult to obtain reliable information from children with severe intellectual impairments or significant communication problems [10]. In this sense, some authors have suggested the possibility to assess children’s HRQOL and pain by using reports from proxies [parents, health professionals, and teachers] [18]. Indeed, several studies have used parents’ reports to assess HRQOL and pain prevalence in pediatric population with CP [3,6]. Nevertheless, little is known about health professionals’ estimations of pain and HRQOL in children with CP, despite its importance on treatment choices, patient-doctor relationship, and psychosocial pain management during healthcare-related pain-inducing procedures [12,19-22].

The aim of the present study was to analyze health professionals’ perceptions of pain and HRQOL in children and adolescents with CP, as well as to explore the impact of pain on HRQOL as perceived by health professionals. Based on previous work indicating that pain may play a key role in HRQOL in children with CP, we hypothesized that physiotherapists would rate participants with pain as more impaired in the physical, emotional and social domains of HRQOL than participants without pain. Moreover, consistent with previous results showing that pain is more frequent in children with poorer physical conditions [2,6,23] and that recurrent musculoskeletal pain is associated with reduced HRQOL [9], we further explored physiotherapists’ estimations of pain and HRQOL in individuals with CP and different levels of motor function.

## Method

### Design and procedure

This cross-sectional study was carried out between September 2011 and February 2012. Physiotherapists were initially contacted by one of the authors to explain the details of the study. Physiotherapists who were treating children and adolescents with CP on a regular schedule (at least one 45-minute session during 2–3 days/week) were asked to participate in the study and to answer several questionnaires concerning pain, HRQOL, level of motor function and demographic data for each individual with CP. Inclusion criteria were: 1) individuals with CP should be treated by physiotherapists at least during the previous six months, and 2) age of individuals with CP should be between 8 and 19years old. Parental informed written consent was obtained for all individuals with CP. The study was approved by the Ethics Committee of University of Salamanca (Spain).

### Participants

A convenience sample of children and adolescents with CP and their physiotherapists were recruited from associations for the care of individuals with cerebral palsy and related disabilities (ASPACE) in Balearic Islands and Castile Leon (Spain). Forty-five physiotherapists treating this sample were contacted, and 35 of them (78%) decided to participate in the study. Physiotherapists’ reports from 91 individuals with CP were obtained. The sample of individuals with CP was composed of girls (54.9%) and boys (45.1%) with a mean age of 12years old (range between 8 and 19years).

### Measures

Physiotherapists completed an interview-based protocol with items about sociodemographic and clinical characteristics (pain, motor function and health-related quality of life) of individuals with CP. Physiotherapists were asked to assess individual’s behavior by direct observation of participants.

#### Bodily pain and discomfort

Physiotherapists reported presence and severity of pain in individuals with CP by using the SPARCLE [Study of Participation of Children with Cerebral Palsy Living in Europe] protocol [24]. This protocol assesses pain with two items of the domain Bodily Pain and Discomfort from the Child Health Questionnaire [25]. In this questionnaire, pain was evaluated by two items: 1) a yes/no question about the presence of physical pain in the last 4weeks, and 2) a question about pain intensity (0 = none, 1 = low, 2 = moderate or 3 = severe pain).

#### Gross motor function classification system (GMFCS)

Physiotherapists completed the GMFCS to classify the level of motor function of their patients with CP [26]. This instrument classifies functional mobility into five levels, with level I indicating the highest motor function independence and level V, the lowest gross motor function.

#### Health-related quality of life (HRQOL)

Physiotherapists assessed health-related quality of life (HRQOL) in children and adolescents with CP by using the Spanish version of the KIDSCREEN [17]. This questionnaire provides information of HRQOL through 52 items grouped into10 dimensions: Physical Well-being (5 items), Psychological Well-being (6 items), Moods and Emotions (7 items), Self-Perception (5 items), Autonomy (5 items), Parent Relations and Home Life (6 items), Social Support and Peers (6 items), School Environment (6 items), Social Acceptance (Bullying) (3 items), and Financial Resources (3 items). Cronbach’s alpha values are greater than .70 for all dimensions [17]. The questionnaire includes a version for children and adolescents aged between 8 and 18 years and a version for parents/proxy. The proxy version of this questionnaire was applied in the present study. Raw scores were transformed to T-scores (mean = 50 and standard deviation = 10) with higher values indicating higher HRQOL.

### Data analysis

Spearman correlations were computed to test the relationship between different domains of HRQOL and levels of motor function, as well as pain intensity. Statistical analysis was performed with SPSS v.19. The significance level was set at p < .05.

## Results

Table 1 displays sociodemographic and clinical characteristics of individuals with CP. Most participants had bilateral spastic diplegia (62.6%) with high levels (IV-V) of motor dysfunction (51.6%), moderate to severe cognitive impairment (55%), and communication problems (59.3%), visual disability (48.4%), behavioural problems (41.8%) and epilepsy as major comorbidities (Table 1).

**Table 1:** **Clinical and sociodemographic characteristics of children and adolescents with cerebral palsy (*n =*** **91) assessed by physiotherapists (n = 35)**

***Clinical variable***	***N***	***%***
*Gender*		
Male	41	45.1
Female	50	54.9
*Type of cerebral palsy*		
Bilateral spastic	57	62.6
Unilateral spastic	10	11.0
Other (diskinetic, ataxic, mixed)	24	26.4
*Pain present in the last 4weeks*		
Yes	38	51.4
No	36	48.6
*Pain intensity*		
None	36	50.0
Mild	22	30.6
Moderate	9	12.5
Severe	5	6.9
*Motor impairment (GMFCS)*		
Level I	17	18.7
Level II	20	22.0
Level III	7	7.7
Level IV	10	11.0
Level V	37	40.6
*Cognitive impairment*		
None	10	11.0
Mild	31	34.0
Moderate	22	24.2
Severe	28	30.8
*Comorbidities*		
Epilepsy	38	41.8
Hearing disability	7	7.7
Visual disability	44	48.4
Behavior problems	38	41.8
Communication problems	54	59.3
Feeding problems	31	34.1
*Type of schooling*		
Regular school	25	27.5
Special school	63	69.3
Both	3	3.2
*Technical aids and services*		
Wheelchair	46	50.5
Orthopedic	39	42.9
Communication	27	29.7

Physiotherapists reported that pain (defined as persistent physical pain perceived during the last four weeks) was present in 51.4% of children and adolescents with CP. Moreover, pain was perceived as mild in 61.1%, as moderate in 25.0%, and as severe in 13.9% of participants. Table 2 displays physiotherapists’ ratings (standardized T-scores) of health-related quality of life (HRQOL) in individuals with cerebral palsy. Physiotherapists estimated that most domains of HRQOL in individuals with CP were within one standard deviation below the mean (all T-scores > 40) as compared with normative data. In the case of physical well-being, group average was more than one standard deviation below the mean (T-score = 38.4).

**Table 2:** Mean **and SD of standardized T-scores on health-related quality of life domains in individuals with cerebral palsy (CP) as assessed by physiotherapists**

***Domains***	***n***	***Mean (SD)***
Physical well-being	59	38.4 (7.8)
Psychological well-being	70	46.3 (9.9)
Moods and emotions	59	48.2 (9.4)
Self-perception	52	46.6 (8.2)
Autonomy	61	44.1 (11.1)
Parent relations and home life	53	47.4 (10.6)
Social support and peers	51	43.7 (10.2)
School environment	76	51.7 (6.5)
Social acceptance (bullying)	75	48.3 (10.0)
Financial resources	33	46.6 (13.2)

Significant negative correlations were obtained between physiotherapists’ estimations of pain intensity and HRQOL domains such as psychological well-being and school environment (Table 3), indicating that high levels of pain were associated with lower HRQOL in these domains. Significant correlations were also found among physiotherapists’ estimations of motor impairment and physical well-being, autonomy and social acceptance domains of the HRQOL. In the case of physical well-being and autonomy, physiotherapists considered that impaired motor function was associated with reduced levels of HRQOL in these domains, whereas impaired motor function was related to high social acceptance. No significant correlation was found between physiotherapists’ estimations of level of motor impairment and pain intensity.

**Table 3:** Spearman correlations between physiotherapists’ estimations of health-related quality of life (higher scores = best health-related quality of life), level of motor function (higher scores = worst motor function) and pain intensity (higher scores = most severe pain) in individuals with cerebral palsy

***Domains***	***Level of motor function***		***Pain intensity***	
	***n***		***n***	
Physical well-being	51	-.40**	50	-.23
Psychological well-being	63	-.13	59	-.27*
Moods and emotions	52	.08	52	-.15
Self-perception	45	.03	45	-.05
Autonomy	53	-.23*	51	.07
Parent relations and home life	47	-.05	44	-.13
Social support and peers	44	-.16	44	.23
School environment	67	.03	62	-.27*
Social acceptance (bullying)	67	.29**	56	.20
Financial resources	30	-.11	27	-.12

*p < .05.

**p < .01.

## Discussion

The aim of the present study was to analyze physiotherapists’ perceptions of pain and HRQOL in individuals with CP. Physiotherapists estimated that more than half of individuals with CP were suffering from pain of mild intensity. This finding was in agreement with previous research indicating that persons with CP experience persistent pain [4,27] on a daily or weekly basis [1,3-5]. Thus, this data may suggest that physiotherapists can accurately recognize pain in their patients with CP. Given that pain symptoms make children with neurological impairments especially vulnerable to poor pain management [28], the adequate recognition of pain in individuals with CP by physiotherapists was of paramount importance.

We also found that health professionals associated pain with significant reductions of positive emotions and life satisfaction in children and adolescents with CP. This was also in agreement with previous studies with self-reports and proxy-reports from parents showing that pain affected emotional and psychological well-being in individuals with CP [6,10-12,18,29]. A relevant finding of the present study was that physiotherapists considered that pain in cerebral palsy was not associated with low HRQOL scores in the physical domain. This finding differed from data obtained from children and parents’ reports, showing significant correlations between pain and physical components of quality of life and mobility [10,23]. This disagreement between physiotherapist and patients’ perception of pain consequences has been also shown by studies in other situations, such as pain induced by mobilizations [30], where health professionals emphasized the positive over the negative dimensions of procedural pain. Moreover, these findings underlined the importance of psychosocial factors as predictors of pain, as well as the role of biopsychosocial models for understanding chronic pain in persons with CP [31].

On the other hand, although physiotherapists reported no significant relationship between motor disability and pain, increased motor impairment was related to low physical well-being and limited autonomy. Our data seems to be in agreement with previous reports [10,14,18]. Thus, for instance, high motor impairment has been associated with lower physical activity, energy and fitness and lower self-determination in individuals with CP [10,14,18,32]. Furthermore, previous studies analyzing professionals’ reports have shown that the severity of child’s physical impairment is associated with lower quality of life in physical well-being and autonomy domains of the KIDSCREEN questionnaire [18]. In addition, our results suggested that impairments in gross motor function could be linked to a lower risk of having poor HRQOL in social acceptance domains. A possible explanation of this result could be that children with CP are less frequently in contact with their non-disabled peers [10] and, therefore, they might have less possibilities of feeling rejected or harassed.

Furthermore, health professionals perceived that enhanced pain was associated with reduced life satisfaction and negative feelings towards school in individuals with CP. This finding was in agreement with previous data from parents and self-reports showing that severe pain in children with spastic CP also led to impaired school performance [8]. Furthermore, it has been shown that severe pain could be a determinant of psychological adjustment, and that emotional symptoms may be significantly increased with the presence of severe pain in CP [33]. A comprehensive assessment of pain in CP would require documenting pain history (e.g., presence of surgical interventions) and collecting information about biopsychosocial factors (e.g. anxiety, depression) which could be relevant for the maintenance of pain over time. An appropriate recognition of pain by physiotherapist, together with a comprehensive assessment of pain may allow health professionals to provide adequate coping strategies for better pain management in children and adolescents with cognitive impairments and poor communication abilities, such as individuals with CP. In this sense, our findings specifically addressed the importance of HRQOL and pain assessment carried out by physiotherapists who often treat individuals with CP on a regular schedule and are often the first caregiver to notice a change in HRQOL and pain. Nevertheless, it should be noted that parents and health professionals may have different perceptions of child’s HRQOL and pain in several chronic diseases (e.g., leukaemia, juvenile idiopathic arthritis, asthma or cystic fibrosis) [34]. Thus, our study suggested that, whenever possible, multi-respondent assessment of HRQoL and pain should be considered in individuals with cerebral palsy.

This study has some limitations that must be taken into account for interpretation of results. The analysis of pain and health-related quality of life in children and adolescents with cerebral palsy was based only on surrogate pain reports provided by their physiotherapists. Nevertheless, a combination of self-reports and proxy-reports from parents, caregivers and health professionals would have provided a better understanding of pain and health-related quality of life in this population. Although our sample of individuals with CP was selected from educational settings in different Spanish regions, sample size and response rates were low. Moreover, our sample of children and adolescents with CP displayed different levels of cognitive impairment (ranging from none to severe), which may have differentially influenced health professionals’ perception about their pain and health-related quality of life. The cross-sectional design of the present study represents a further limitation, as it can be used only to describe pain and HRQOL characteristics that exist in the population of children and adolescents with CP, but not to determine cause-and-effect relationships among these variables and other relevant factors as mobility, cognitive impairment or type of CP. Nevertheless, although our study does not provide information about the influence of pain on HRQOL over time in CP, it lays a scientific basis for the implementation of a longitudinal design and guides the selection of appropriate outcome measures for future studies.

## Conclusions

In conclusion, health professionals perceived a high prevalence of pain in their patients with CP. Physiotherapists also reported that pain might lead to significant reductions on psychological well-being and school domains of HRQOL. Moreover, they considered that motor impairment rather than pain could be relevant for the reduced quality of life in physical domain observed in children and adolescents with CP. Our findings further underline the relevance of physiotherapist’s view about pain and HRQOL in pediatric patients with CP. More specifically, physiotherapists have a particularly unique view about pain and health-related quality of life in their CP patients and may be the first to report and intervene for pain. These results may contribute to incorporate standard measures for the assessment and design of therapeutic interventions and to guide pain management in persons with CP through their life span.

## Abbreviations

CP: Cerebral palsy; HRQOL: Health-related quality of life; ASPACE: Associations for Care of People with Cerebral Palsy and Related Disabilities; SPARCLE: Study of Participation of Children with Cerebral Palsy Living in Europe; GMFCS: Gross motor function classification system.

## Competing interests

The authors declare that they have no competing interests.

## Authors’ contributions

MB conceived of the study, participated in its design and coordination, participated in the performance of the statistical analysis and helped to draft the manuscript. PM participated in the design of the study, participated in the performance of the statistical analysis and revised the manuscript draft. IR and LA participated in the design of the study, collected data, participated in statistical analysis and helped to draft the manuscript. BO and RA participated in statistical analysis and helped to draft the manuscript. All authors read and approved the final manuscript.

## Pre-publication history

The pre-publication history for this paper can be accessed here:

http://www.biomedcentral.com/1471-2431/14/192/prepub
